# Personality traits and the degree of work addiction among Polish women: the mediating role of depressiveness

**DOI:** 10.3389/fpubh.2023.1305734

**Published:** 2023-12-20

**Authors:** Kamila Rachubińska, Anna Maria Cybulska, Ewa Kupcewicz, Mariusz Panczyk, Szymon Grochans, Ireneusz Walaszek, Elżbieta Grochans

**Affiliations:** ^1^Department of Nursing, Faculty of Health Sciences, Pomeranian Medical University in Szczecin, Szczecin, Poland; ^2^Department of Nursing, Collegium Medicum, University of Warmia and Mazury in Olsztyn, Olsztyn, Poland; ^3^Department of Education and Research in Health Sciences, Faculty of Health Science, Medical University of Warsaw, Warsaw, Poland; ^4^Department of Pediatric and Oncological, Urology and Hand Surgery, Pomeranian Medical University in Szczecin, Szczecin, Poland

**Keywords:** personality traits, workaholism, depression, behavioral addiction, public health

## Abstract

**Objectives:**

Workaholism is an addiction, however the obsessive-compulsive components alone may prove insufficient in determining its nature. The aim of the following study was to determine the mediating role of depressiveness in the relationships between workaholism and personality traits according to the five-factor model among Polish women.

**Methods:**

The research study was carried out among 556 women residing in the West Pomerania Voivodeship in Poland. The research was based on a survey performed using a questionnaire technique. The following research instruments adapted to Polish conditions were employed to assess the incidence of work addiction among female adults: The NEO Five-Factor Inventory (NEO-FFI), The Work Addiction Risk Test (WART) Questionnaire, and The Beck Depression Inventory–BDI I-II.

**Results:**

A positive correlation between the intensity of neuroticism and the work addiction risk was revealed (*β* = 0.204, *p* < 0.001). A partial mediation (35%) with the severity of depression symptoms as a mediating factor was observed (*β* = 0.110, *p* < 0.001). Respondents characterized by high neuroticism showed a greater severity of the symptoms of depression (*β* = 0.482, *p* < 0.001), which is a factor increasing the work addiction risk (*β* = 0.228, *p* < 0.001). No effect of extraversion intensity on the work addiction risk was found (*β* = 0.068, *p* = 0.081). Respondents characterized by a high level of extraversion displayed lower severity of the symptoms of depression (*β* = −0.274, *p* < 0.001). A negative correlation between the intensity of agreeableness and the work addiction risk was revealed (*β* = −0.147, *p* < 0.001). A partial mediation (27.8%) was observed. A positive correlation between the intensity of conscientiousness and the work addiction risk was revealed (*β* = 0.082, *p* = 0.047). Respondents characterized by a high level of conscientiousness showed a lower severity of depression symptoms (*β* = −0.211, *p* < 0.001).

**Conclusion:**

Depressiveness plays the role of a mediator between neuroticism, extraversion, agreeableness as well as conscientiousness, and work addiction. Depressiveness is a factor which increases the risk of work addiction.

## 1 Introduction

The workaholism phenomenon is one of the social problems recognized as significant in the modern world. In most studies, it is defined as a multidimensional occurrence which includes the following components: behavioral, cognitive and affective (1–3). Work addiction is oftentimes defined as spending undue amounts of time at work, exceeding the alleged norms, as well as being preoccupied with work (4). This definitional approach is supported by research (5). The model proposed by Salomon recognizes workaholism as a type of addiction. It states that addiction is when: work awakens a pleasantly emotional state, affective tolerance arises from the need to increase workload, and signs of abstinence are recognized in cases of work being prevented. This model indicates that the compulsion to work evokes positive emotions, which in turn reinforces certain types of behavior (3, 6). Seligman presents another concept of workaholism, believing that it is a specific way of functioning of an employee caused by work addiction (6). One of its aspects is a strong commitment to work, which is viewed positively by employers. However, such an assessment may concern the initial phase of workaholism development only. In the long term, the workaholism phenomenon should be assessed negatively. Like any addiction, it has detrimental effects on the individual and the organization that employs them (7, 8). Other threats to health and mental functioning of the work addict are also recognized, such as excessive and long-term stress or professional burnout related to work performance. Therefore, maintaining the right balance between professional responsibilities and one’s relaxation time, effective ways of handling difficult situations, as well as ‘healthy’ attitude towards work and one’s job position, all seem to be essential in maintaining mental hygiene and well-being (9).

The knowledge of this phenomenon, its mechanisms and consequences remains incomplete. It has not been listed in either, the ICD-10 or DSM-5 classifications (10). The relationship between an individual and their work is defined by three dimensions: work commitment, feeling driven to work, and pleasure from work (2). Various compilations of these dimensions construct the employee type. Workaholics are very preoccupied with work, they feel a compulsion to do it, but they do not derive satisfaction from it (11, 12). On the other hand, work enthusiasts experience satisfaction from work whilst not feeling the internal pressure to perform it. There are many factors contributing to the occurrence of this type of behavioral addiction that have not yet been thoroughly investigated, i.e. personality traits (11).

Personality is a set of emotional and behavioral characteristics which accompany an individual in their daily life (13). Researchers believe that the individual’s personality traits are related to their type of work and chosen profession (14, 15). The personality model known as The Big Five, proposed by Costa and McCrea (16), includes personality traits such as Neuroticism, Extraversion, Openness to experience, Agreeableness and Conscientiousness (17). Burke et al. (18) believe that work addiction can be analyzed on the basis of personality traits. Studies have shown that there is a positively strong correlation between high work willingness scores and neuroticism. Work commitment and enjoyment of work have been confirmed to have a positive relationship with extraversion, as extroverts tend to be energetic and sociable, as well as have a positive impact on success. Neuroticism determines the internal pressure which causes an increase in anxiety and constant thinking about work, however it is assumed that work will alleviate this undesirable feeling (19). Conscientiousness is positively associated with all dimensions of work addiction (18).

Wojdylo, who has taken up the concept of a workaholic personality, informs that it is characterized by a compulsion to work and being overloaded with work (20). The workaholics are characterized by a high level of extrinsic motivation (e.g., the need for social approval) and a high level of motivation to avoid failure (avoidance orientation) (21). The mechanism of work obsession is involved in this process, manifested in, i.e., above-average expenditure of energy at work, overstating the standards of task performance, certain repetitiveness of action and the inability to “switch off.” In addition, an important role is played by fear-influenced negative emotions, as well as the so-called mobilizing stimulation. Other factors of high significance in the development and perpetuation of work addiction are: low emotional self-determination (AUTEM) and high intellectual self-determination (AUTIN) (20, 22). Available research shows that work addicts showed higher levels of aspiration, worked comparably persistently and were not significantly different from non-workaholics in terms of work achievement. The data obtained have confirmed the thesis that workaholism is related to the motivation of avoiding failure (20, 22).

Another predictor of the occurrence of behavioral addictions is depressiveness (23–25). To date, few studies have examined the mechanisms underlying the relationship between workaholism and mental health problems such as depression. Depression appears to be significantly associated with workaholism, leading directly to work addiction (and vice versa) (26, 27). Depression seems significantly connected to work addiction. A study by Yang et al. (28) found positive associations between workaholism and depression. The link between workaholism and depression has been shown in studies conducted in various professional and cultural contexts (29–32) A cross-sectional survey on a large sample of 16,426 employees showed positive and significant correlations between workaholism and all tested symptoms of mental disorders, including depression (33–37). It is not only workaholics who suffer from poor mental health as a result of addiction. A study comparing adult children of workaholics and non-workaholics found that children of workaholics had higher levels of depression (38).

Recent studies have revealed that the relationship between workaholism and depressiveness was stronger among women than among men (11). Biological, social, and psychological factors contribute to the gender-based differentiation in the occurrences of depression (12). Depression is 2–3 times more common in women than in men (39, 40). The neurobiological and endocrine systems predispose women to experience more intense and persistent feelings of anger and depression than men (41). According to research by Chuick et al. (42), men differ from women on the basis of experiencing depression. While men externalize their depression, women tend rather to internalize it (43–47).Women have two to three times higher risk of experiencing depression. The difference between genders in the occurrence of depression has been confirmed in numerous studies across various cultures and age groups, including undergraduate students. Although there are also conflicting studies, female gender remains one of the most consistent risk factors for depression (48, 49). We recognized the need to fill this research gap. Therefore, the authors of the following study decided to conduct research among the female population only.

Theories of psychological stress explain that everyday stressors (e.g., personality traits, daily stressful events) can cause acute or long-term psychological distress, which in turn contributes to the occurrence of depressive symptoms (31). The basic element of workaholism, obsessive-compulsiveness at work, is a strong stressor that can cause work-life conflict and stressful feelings (50). Work addiction and long working hours can limit resources (e.g., cognitive, energy, time, social) and effort to take care of one’s life. The result is stress in various aspects of life, including sleep problems (51), hostile and ineffective interpersonal relationships (52), poor social functioning (53) and family conflicts (54). In turn, stress related to work and private life may intensify depressive symptoms. Empirical studies have shown significant positive associations between workaholism and work–family conflict (55) and between work–family conflict and depression (28).

Undoubtedly, workaholism is an addiction, however the obsessive-compulsive components alone may prove insufficient in determining its nature. Workaholism has three dimensions: behavioral, cognitive, and affective (56). There are studies available on, for example, personality traits and workaholism, personality traits and depressive or depressive and workaholism. However, there is a lack of research that takes into account several factors at once, such as depressive and personality traits. To the best of our knowledge, this is the first study that examines the relationships between depressiveness, personality traits and workaholism among women in a single sample. Our findings are important because they shed new light on the relationship between workaholism, personality traits and depression. The relationship between personality traits, depression, and work addiction has been extensively discussed in many studies, but is a scarcity of studies focusing on the female population, which is why we have undertaken deliberations on this subject. We acknowledged the necessity to address this gap and described this aspect in the limitations section. Women have two to three times higher risk of experiencing depression. The difference between genders in the occurrence of depression has been confirmed in numerous studies across various cultures and age groups. Although there are also conflicting studies, the female gender remains one of the most consistent risk factors for depression. We recognized the need to fill this research gap. Further research within this specific field is necessary. The aim of the following study was to determine the mediating role of depressiveness in the relationships between workaholism and personality traits according to the five-factor model called The Big Five among Polish women. The main research problem was formulated in a form of a question: Does depressiveness mediate the relationship between personality traits and the occurrence of work addictions among Polish women? Hypothesis 1: Depression will be a significant mediator of the relationship between neuroticism and the level of work addiction. Hypothesis 2: A significant mediator of the relationship between extraversion and the work addiction will be depression. Hypothesis 3: The relationship between openness to experience and work addiction will not be mediated by depression. Hypothesis 4: In the mediation model depression will not be any significant mediator between Agreeableness Predictor and work addiction. Hypothesis 5: Depression will be a mediator in the mediation model between conscientiousness and work addiction.

## 2 Materials and methods

### 2.1 Settings and design

The study involved 556 women from the West Pomerania Voivodeship in Poland. When selecting the sample for the study, the sample size calculator of the statistical program STATISTICA version for Windows 13.1 TIBCO Software Inc. was used. – StatSoft, Poland, with 95% confidence interval. This made the sample representative. Based on data on the number of women living in the West Pomeranian Voivodeship, the minimum group of patients that should be included in the study is 384 people. The selection of the group was random. No randomizing tool was used. It was based on self-reported participants who met the inclusion criteria as described in the limitations. The criteria for inclusion in the study were: female sex, age ≥ 18 years, place of residence in the West Pomerania Voivodeship, no self-reported psychiatric diseases, giving informed written consent to participate in the study, and completion of the questionnaires. The respondents were familiarized with the aim of the research and informed about the possibility of withdrawing from the study at any stage. This study is a part of a larger project concerning the incidence of behavioral addictions among women. It was approved by the Bioethics Committee of the Pomeranian Medical University in Szczecin (KB-0012/518/12/16) and conducted in accordance with the Declaration of Helsinki (57).

### 2.2 Research instruments

The study was based on a survey performed using a questionnaire technique.

#### 2.2.1 The NEO Five-Factor Inventory (NEO-FFI)

The NEO Five-Factor Inventory (NEO-FFI) is a standardized instrument for analyzing personality traits included in Costa and McCrea’s five-factor model, known as The Big Five. It is divided into five subscales measuring: neuroticism, extraversion, openness to experience, agreeableness, and conscientiousness. Each subscale contains 12 statements rated by the respondent on a five-point scale (from 0 to 4 points). In some cases the direction of scoring is reversed. The possible raw scores range from 0 to 48 points, and are converted into sten scores. The higher the score on a given subscale, the greater the intensity of a given trait (58, 59).

#### 2.2.2 The Work Addiction Risk Test (WART)

A questionnaire developed by Robinson and Phillips measures the symptoms of a workaholic’s behavior pattern. The Polish version of The Work Addiction Risk Test (WART) was created with the use of the translation procedure. Cronbach’s alpha coefficient (0.87) indicates a satisfactory internal consistency of the questionnaire. The tool consists of 25 statements rated on a four-point scale, measuring the behavioral, cognitive, and emotional responses that are believed to constitute workaholism syndrome. Depending on the score, the questionnaire measures fully formed workaholism syndrome or work addiction risk. The higher the result, the greater the probability of developing workaholism (60–62).

#### 2.2.3 The Beck Depression Inventory–BDI I-II

The Beck Depression Inventory (BDI I-II) is used to self-assess the severity of depressive disorders. It contains 21 questions with four answer options. The final scores are calculated by summing up the points obtained for each question. They reflect the level of depression and are interpreted as follows: 0–13—no depression or minimal symptoms of depression, 14–19—mild depression, 20–28—moderate depression, and 29–63—severe depression (63).

### 2.3 Statistical analysis

Quantitative data collected by means of standardized scales were presented using descriptive statistics parameters. The mean, standard deviation (SD), range (min-max) and skewness were determined. To obtain a symmetric distribution of the variables, the Box-Cox transformation was performed (64), which is a transformation of non-normal variables into a normal shape. Normality is an important assumption in mediation analysis. In order to estimate the effect of the mediator (depressiveness) on the relationship between the independent variable and the dependent variable, The Generalized Linear Model (GLM) mediation analysis. The mediation model was fitted to each sample, resulting in a bootstrap sample. Bootstrapping is a statistical method that uses random resampling with replacement to estimate a population parameter. The technique involves sampling from a given dataset to estimate a parameter when it would otherwise be impossible (65).

The data were analyzed using IBM SPSS v. 28 for descriptive analysis and Jamovi v. 2.2.5 with the jAMM module for testing the mediation model (66). The jAMM module makes it possible to estimate the direct and indirect impact of independent variables on dependent variables, also examining all the paths of the mediation model components (e.g., relationships between independent variables and the mediator and relationships between the mediator and dependent variables) (67). Given that five mediation models were evaluated, the Šidák correction was applied to mitigate inflation of the Type I error rate (68). A statistical significance level of 1% was assumed for all analyzes in rejecting the null hypothesis.

### 2.4 Brief characteristics of the respondents

556 women residing in the West Pomerania Voivodeship participated in the study. The average age of the participants was 34 years. Most of the respondents had lower education (51.6%). Most of the women declared being in a formal/informal relationship (66.5%) and employed (89.2%).

## 3 Results

### 3.1 Analysis of variable values

The data obtained via NEO-FFI Personality Inventory showed that the greatest intensity of personality traits in the studied group of women was demonstrated in the conscientiousness subscale (6.56 ± 2.18). The lowest intensity of characteristics was found in the neuroticism subscale (5.82 ± 2.33). The WART questionnaire measured the symptoms of workaholism. The respondents achieved an average score of 53.46 ± 12.24 points. The Beck Depression Inventory – BDI I-II measured the symptoms of depression. The mean score was 6.8 ± 7.345 (Table 1).

**Table 1:** Description of the primary variables and the after Box-Cox transformation for *n* = 557.

Variable	M	SD	Mdn	IQR/2	Min – Max	CV [%]	Skewness
N stens	5.82	2.33	6.00	1.50	1.00–10.00	40.07	0.04
E stens	6.22	2.15	6.00	1.50	1.00–10.00	34.55	−0.14
O stens	5.60	1.98	5.00	1.50	1.00–10.00	35.32	0.18
A stens	6.18	2.21	6.00	1.50	1.00–10.00	35.68	−0.02
C stens	6.56	2.18	7.00	1.50	0.00–10.00	33.17	−0.42
WART scoring	53.46	12.24	53.00	8.50	25.00–95.00	22.90	0.24
WART scoring*	19.25	3.17	19.26	2.19	11.14–29.01	16.46	0.00
BDI scoring	6.80	7.35	4.50	4.50	0.00–40.00	108.01	1.69
BDI scoring*	1.78	1.17	1.84	0.98	0.00–4.43	65.44	−0.03

M, mean; SD, standard deviation; Mdn, median; IQR, semi-quartile range; Min, minimum; Max, maximum; CV, coefficient of variation; N, Neuroticism; E, Extraversion; O, Openness to experience; A, Agreeableness; C, Conscientiousness; WART, Work Addiction Risk Test; BDI, Beck Depression Inventory * Box-Cox transformation.

### 3.2 Mediation analysis

A mediation analysis was carried out to determine whether depression is a significant mediator of the relationship between personality traits (NEO-FFI) and the level of work addiction (WART). The results revealed statistically significant mediations.

#### 3.2.1 Mediation model 1: neuroticism predictor

A positive correlation between the intensity of neuroticism and the work addiction risk was revealed (*β* = 0.204, *p* < 0.001). A partial mediation (35%) with the severity of depression symptoms as a mediating factor was observed (*β* = 0.110, *p* < 0.001). Respondents characterized by a high level of neuroticism showed a greater severity of the symptoms of depression (*β* = 0.482, *p* < 0.001), which is a factor increasing the work addiction risk (*β* = 0.228, *p* < 0.001) (Table 2).

**Table 2:** Indirect and total effects: mediation model 1 – neuroticism.

			95% CI*				
Type	Effect	b	Lower	Upper	β**	z	*p*-value
Indirect	N ⇒ BDI ⇒ WART	0.149	0.092	0.213	0.110	4.800	<0.001
Component	N ⇒ BDI	0.241	0.205	0.275	0.482	13.270	<0.001
	BDI ⇒ WART	0.618	0.398	0.851	0.228	5.230	<0.001
Direct	N ⇒ WART	0.277	0.157	0.403	0.204	4.540	<0.001
Total	N ⇒ WART	0.426	0.319	0.534	0.314	7.790	<0.001

NEU, neuroticism; WART, Work Addiction Risk Test; N, Neuroticism; BDI, Beck Depression Inventory–BDI I-II; b, unstandardized regression coefficient; *β*, standardized regression coefficient; *p*, significance level; * Confidence interval (CI) computed with method: bootstrap percentiles; ** Beta (*β*) is completely standardized effect size.

#### 3.2.2 Mediation model 2: extraversion predictor

No effect of extraversion intensity on the work addiction risk was found (β = 0.068, *p* = 0.081). A complete mediation (58.3%) with the severity of depressive symptoms as a mediating factor was observed (β = −0.094, *p* < 0.001). Respondents characterized by a high level of extraversion displayed lower severity of the symptoms of depression (β = −0.274, *p* < 0.001), which is a factor increasing the work addiction risk (β = 0.345, *p* < 0.001) (Table 3).

**Table 3:** Indirect and total effects: mediation model 2 – extraversion.

			95% CI*				
Type	Effect	b	Lower	Upper	β**	z	*p*-value
Indirect	E ⇒ BDI ⇒ WART	−0.139	−0.194	−0.089	−0.094	−5.083	<0.001
Component	E ⇒ BDI	−0.148	−0.192	−0.106	−0.274	−6.726	<0.001
	BDI ⇒ WART	0.936	0.705	1.169	0.345	7.902	<0.001
Direct	E ⇒ WART	0.099	−0.005	0.216	0.068	1.744	0.081
Total	E ⇒ WART	−0.040	−0.162	0.083	−0.027	−0.632	0.527

EKS, extraversion; WART, Work Addiction Risk Test; E, Extraversion; BDI, Beck Depression Inventory–BDI I-II; b, unstandardized regression coefficient; *β*, standardized regression coefficient; *p*, significance level; * Confidence interval (CI) computed with method: bootstrap percentiles; ** Beta (*β*) is completely standardized effect size.

#### 3.2.3 Mediation model 3: openness to experience predictor

No effect of the intensity of openness to experience on the work addiction risk was found (*β* = 0.042, *p =* 0.263). A mediation with the severity of depressive symptoms as a mediating factor was not observed (*β* = −0.007, *p* = 0.625) (Table 4).

**Table 4:** Indirect and total effects: mediation model 3 – openness to experience.

			95% CI*				
Type	Effect	b	Lower	Upper	β**	z	*p*-value
Indirect	O ⇒ BDI ⇒ WART	−0.011	−0.055	0.036	−0.007	−0.489	0.625
Component	O ⇒ BDI	−0.013	−0.059	0.041	−0.022	−0.492	0.623
	BDI ⇒ WART	0.888	0.668	1.099	0.327	8.072	<0.001
Direct	O ⇒ WART	0.067	−0.050	0.186	0.042	1.120	0.263
Total	O ⇒ WART	0.056	−0.078	0.189	0.035	0.818	0.414

OTW, openness to experience; WART, Work Addiction Risk Test; O, Openness to experience; BDI, Beck Depression Inventory–BDI I-II; b, unstandardized regression coefficient; *β*, standardized regression coefficient; *p*, significance level; * Confidence interval (CI) computed with method: bootstrap percentiles; ** Beta (*β*) is completely standardized effect size.

#### 3.2.4 Mediation model 4: agreeableness predictor

A negative correlation between the intensity of agreeableness and the work addiction risk was revealed (*β* = −0.147, *p* < 0.001). A partial mediation (27.8%) with the severity of depression symptoms as a mediating factor was observed (*β* = −0.056, *p* < 0.001). Respondents characterized by a high level of agreeableness showed a lower severity of the symptoms of depression (*β* = −0.189, *p* < 0.001), which is a factor increasing the work addiction risk (*β* = 0.298, *p* < 0.001) (Table 5).

**Table 5:** Indirect and total effects: mediation model 4 – agreeableness.

			95% CI*				
Type	Effect	b	Lower	Upper	β**	z	*p*-value
Indirect	A ⇒ BDI ⇒ WART	−0.081	−0.130	−0.040	−0.056	−3.490	<0.001
Component	A ⇒ BDI	−0.100	−0.150	−0.050	−0.189	−4.100	<0.001
	BDI ⇒ WART	0.811	0.583	1.058	0.298	6.760	<0.001
Direct	A ⇒ WART	−0.211	−0.330	−0.088	−0.147	−3.420	<0.001
Total	A ⇒ WART	−0.291	−0.408	−0.174	−0.203	−4.880	<0.001

UGD, agreeableness; WART, Work Addiction Risk Test; A, Agreeableness; BDI, Beck Depression Inventory–BDI I-II; b, unstandardized regression coefficient; *β*, standardized regression coefficient; *p*, significance level; * Confidence interval (CI) computed with method: bootstrap percentiles; ** Beta (*β*) is completely standardized effect size.

#### 3.2.5 Mediation model 5: conscientiousness predictor

A positive correlation between the intensity of conscientiousness and the work addiction risk was revealed (*β* = 0.082, *p* = 0.047). A partial mediation (47%) with the severity of depressiveness symptoms as a mediating factor was observed (*β* = −0.072, *p* < 0.001). Respondents characterized by a high level of conscientiousness showed a lower severity of depression symptoms (*β* = −0.211, *p* < 0.001), which is a factor increasing the work addiction risk (*β* = 0.343, *p* < 0.001) (Table 6).

**Table 6:** Indirect and total effects: mediation model 5 – conscientiousness.

			95% CI*				
Type	Effect	b	Lower	Upper	β**	z	*p*-value
Indirect	C ⇒ BDI ⇒ WART	−0.105	−0.168	−0.054	−0.072	−3.670	<0.001
Component	C ⇒ BDI	−0.113	−0.165	−0.063	−0.211	−4.400	<0.001
	BDI ⇒ WART	0.933	0.707	1.175	0.343	8.081	<0.001
Direct	C ⇒ WART	0.119	0.004	0.241	0.082	1.982	0.047
Total	C ⇒ WART	0.014	−0.108	0.135	0.009	0.221	0.825

SUM, conscientiousness; WART, Work Addiction Risk Test; C, Conscientiousness; BDI, Beck Depression Inventory–BDI I-II; b, unstandardized regression coefficient; *β*, standardized regression coefficient; *p*, significance level; * Confidence interval (CI) computed with method: bootstrap percentiles; ** Beta (*β*) is completely standardized effect size.

## 4 Discussion

Research on the behavioral addictions have shown that people overly involved in particular activities tend to struggle with problematic social lives and often experience depression (57, 69, 70). Therapists, doctors and researchers are increasingly encountering cases of compulsive behavior―in addition to gambling, more and more often the subject of preoccupation is work addiction (4). As far as we know, ours is the first study to examine the mediating role of depressiveness in the relationships between workaholism and personality traits.

Anxiety and depression are mental health problems that can increase the risk of addiction (33, 71–73). The link between depression and work addiction have been already reported in many studies (73–78). It is also known that workaholism may result from an attempt to reduce the feeling of anxiety and depression.

In the modern society, hard work is praised and honored, thus serving as an individual’s legitimate behavior aimed at combatting or mitigating negative feelings – as well as improving one’s well-being and self-esteem (79).

Using the Work Addiction Risk Test (WART) on the Polish population, we relied on the Polish adaptation of WART and its psychometric properties analysis by Wojdyło. According to Wojdyło, the questionnaire measures a fully developed workaholism syndrome or the risk of work addiction, depending on the score level. A score above 56 points indicates a fully developed work compulsion: a high score (67–100 points) indicates a high level of addiction, while a moderate score (57–66 points) indicates a moderate level of addiction. A low score ranging from 25 to 56 points indicates the absence of addiction or the degree of risk for work addiction (the higher the score, the greater the likelihood of developing workaholism). During the validation process, it was assumed that the factors representing the five specific dimensions of workaholism should be correlated and form a superfactor, a common factor (workaholism). Therefore, the factor axes were rotated to a simple structure obliquely using the technique of hierarchical factor analysis. Oblique rotation was also applied because it does not enforce orthogonality between variable clusters, allowing for the possibility of factor correlation and obtaining a simple structure. The analysis of the factor correlation matrix revealed that the factors were related (non-orthogonal), suggesting the presence of a latent common factor (60–62).

In recent years, personality traits have become an extremely interesting topic for researchers in the social and health sciences. The average WART score was 53.46 points, where a score of 25–56 points indicated a low risk of workaholism. However, the higher the score, the higher the risk of addiction. The main study findings revealed partial mediation, depressiveness was recognized as a mediator between neuroticism and the degree of work addiction. The increase in the level of depressiveness resulted in an increase in the degree of work addiction. Authors’ own research revealed that people with a high level of neuroticism showed a higher intensity of depressive symptoms, which is the factor increasing the risk of work addiction. No effect of extroversion intensity on the risk of work addiction was demonstrated. A complete mediation was observed (58.3%) with depressiveness acting as a mediating factor. Respondents with a high level of extraversion showed lower severity of depression symptoms. On the other hand, a negative relationship between the intensity of agreeableness and the risk of work addiction, and the partial mediation (27.8%) were observed. Respondents with a high level of agreeableness characterized by a lower severity of depressive factors. The existence of a positive correlation between the intensity of conscientiousness and workaholism, as well as partial mediation, were demonstrated. Respondents with a high level of conscientiousness showed a lower severity of depression, which increases the risk of work addiction. The analysis of authors’ own research revealed no effect of openness to experience on the risk of work addiction, as well as lack of mediating role of depressiveness. Some key personality traits have a specific influence on work addiction. Individuals who work obsessively tend to exhibit higher levels of conscientiousness and neuroticism. Simultaneously, it seems logical that individuals who are more sensitive to rewards (especially social rewards and recognition) strive to maintain good relationships with others in the workplace (80), thereby showing higher levels of extraversion (81). On the other hand, higher levels of extraversion can be a risk factor for work addiction because individuals with work addiction have a greater need for social feedback from others regarding their achievements, abilities, and success (82). Psychological stress theories (31) explain that daily stressors (such as personality traits) can lead to psychological burden, which, in turn, can contribute to the development of depressive symptoms. The obsessive-compulsive desire for work acts as a significant stressor that can create a conflict between work and personal life and intensify the experience of stress (32). Additionally, work addiction can deplete resources (such as cognitive and social resources).

A meta-analysis by Kun et al. helped elucidate the role of personality factors underlying work addiction. It was found that personality explains only a small portion of the variance in work addiction. Perfectionism, global self-esteem, and negative affect have the strongest associations as personality risk factors for work addiction. Among the Big Five traits, higher levels of extraversion, conscientiousness, and openness to experience contribute to an increased risk of work addiction (83).

Depression was positively associated with workaholism. This finding confirmed the hypothesis and is consistent with previous studies (34–36). Workaholism is often considered a risk factor for depressive symptoms. The link between work addiction and depression has been demonstrated in studies conducted in various professional and cultural contexts (36, 37, 50). According to Haar and Roche (36), both work engagement and job satisfaction are related to anxiety and depression (36). Houlfort et al. (37) assert that depression positively correlates with obsessive passion for work. Similarly, Nie and Sun (35) informed about a noteworthy correlation between work addiction and depression. A cross-sectional study of 16,426 employees demonstrated significant positive correlations between work addiction and all tested symptoms of mental disorders (including depression) (33). The results showed that burnout played a partial mediating role in the relationship between workaholism and depression. Hard work is valued in society, so it can be a legitimate and rationalized behavior of the individual, undertaken to reduce negative feelings, increase their well-being and self-esteem (33). Employers should be aware that workaholism has negative consequences, and understand that overwork does not mean productivity. They should offer employees training in time and stress management, and set standards and values to ensure that both productivity and work-health balance are maintained (84). Research shows the adverse impact of workaholism on health because workaholics work long hours and often do not have the opportunity to regenerate after work, which can cause exhaustion and burnout (85).

Workaholism has both, positive and negative dimensions. Abdolshah et al. (56) showed that workaholism (both dimensions mentioned above) has a significant positive correlation with conscientiousness. On the other hand, the results presented by Burke et al. (18) show that conscientiousness is not significantly related to work addiction. This difference may be related to the characteristics of the sample group, as research demonstrated that people with higher education and high income behave more conscientiously in relation to work, show greater commitment to work and willingness to develop one’s skills (86). Some studies report that agreeableness negatively correlates with drive to work and positively with job satisfaction. Both aspects of workaholism significantly positively correlate with openness to experience. People who care more about new experiences enjoy their jobs more than others. Other results indicate that positive workaholism correlates positively with extraversion. This means that the likelihood of positive workaholism is higher among employees who cultivate social contacts. Both positive and negative workaholism significantly negatively correlates with neuroticism (87).

The relationship between personality traits and workaholism depends on the aspect under consideration. Some researchers look at the subject from a positive point of view and believe that workaholics are satisfied with their work, and thus―productive. In return, researchers with a negative point of perspective think that an increased exposure to overworking is associated with an unpleasant and compulsive phenomenon which hinders the correct daily functioning. Psychological stress theories explain that daily stressors (such as personality traits) can lead to psychological burden, which, in turn, may contribute to the development of depressive symptoms. The obsessive-compulsive desire for work acts as a significant stressor, which can create a conflict between work and personal life and intensify the experience of stress. Furthermore, work addiction can deplete resources (such as cognitive and social resources). As a result, stress can impact psychosocial functioning, including sleep problems, impaired interpersonal relationships, poor social functioning, and family conflicts. Conversely, work-related stress can exacerbate depressive symptoms (56, 88, 89).

Like other emerging behavioral addictions (e.g., the Internet), workaholism is not a substance abuse. Its causes, consequences, and mechanisms have not yet been sufficiently studied. The presented results provide an empirical insight into the social functioning of work addicts. Therefore, further theoretical and empirical research, as well as detailed analyzes of social predictors of work addictions are justified (89–91).

Both current and previous studies (47) may be a starting point for further research on the causes of workaholism. An important goal for mental health professionals dealing with the area of workaholism may be the development of educational guidelines (92).

## 5 Limitations and implications for practice

A major strength of our study was the use of valid and reliable psychometric tools, thanks to which the results significantly enrich the existing literature on behavioral addictions.

Limitations of the study are the sample size, which was not representative, and the fact that only the female population was surveyed. It would be worthwhile to include a male sample in future studies to ensure that the sample size is representative. This limits the possibility of generalization to other populations and excludes the male population. The cross-sectional nature of the study with mediation analyzes based on the parallel data collection does not allow for casual interference. The study was based solely on the self-report measurements of the constructs. Data obtained from interviews may be necessary to present a holistic view of a person’s behavior. The mean score (6.8 ± 7.345) of the Beck Depression Inventory was too low to represent the depression severity of the current sample. According to the cut point, it falls to the level of no depression or minimal symptoms of depression. Another limitation is that we did not analyze other potentially important contributors to behavioral addictions (e.g., stress, insomnia, incomes).

From a theoretical standpoint, our findings are important because they shed new light on the relationship between workaholism and depression. From a practical standpoint, the research presented here may have implications for therapists, as female gender appears to be a risk factor in the depression-pracoholism correlation. Whereas in the light of available evidence, knowledge of the influence of personality traits according to The Big Five model may contribute a strong starting point for further progress in the empirical verification of the causal approach to workaholism. An important goal for mental health professionals working in the area of workaholism could be the development of educational guidelines aimed at softening the approval of materialistic values in order to subsequently minimize the risk associated with particular personality traits, mainly those traits which maintain intermediate effects in this particular phenomenon through materialism (extraversion, openness to experiences, agreeableness and neuroticism) (50).

## 6 Conclusion

The mediation model employed in the study has shown that depressiveness is a mediator between neuroticism, extraversion, agreeableness as well as conscientiousness, and work addiction. Depressiveness is a factor which increases the risk of work addiction. People characterized by a high level of neuroticism show a higher severity of depression and a greater risk of work addiction. People with a high level of extraversion are characterized by less depressive symptoms. Whereas the respondents with high levels of conscientiousness are characterized by a higher risk of workaholism and lower depressiveness. With the increase in agreeableness, the risk of work addiction decreases and the severity of depressive symptoms decreases.

From the basic dimensions of personality according to The Big Five model to the depressiveness, the presented analysis expands knowledge about the risks as well as protective factors of work addiction among women. The results indicate that both types of determinants are necessary and useful for a better understanding of workaholism. Neuroticism and depressiveness constitute risk factors in relation to work addiction. While conscientiousness constitutes a protective factor against said behavioral addiction.

The provided study has practical implications because the obtained results indicate an urgent need to further research personality traits (neuroticism and conscientiousness), as well as the occurrence of depressiveness, which characterizes people addicted to work. The results obtained by the authors of the study encourage the design and implementation of prevention and intervention programs for work addiction, which should work at different levels and enable effective confrontation and management of negative emotions (e.g., anxiety, depression, hostility). Suggestions of this type, among many others, are only intended to awaken the need to ‘take action’ in the face of a growing psychosocial problem in the modern consumer society.

## Data availability statement

The raw data supporting the conclusions of this article will be made available by the authors, without undue reservation.

## Ethics statement

The studies involving humans were approved by Bioethics Committee of the Pomeranian Medical University in Szczecin (KB-0012/46/01/2013). The studies were conducted in accordance with the local legislation and institutional requirements. The participants provided their written informed consent to participate in this study.

## Author contributions

KR: Conceptualization, Data curation, Formal analysis, Methodology, Project administration, Visualization, Writing – original draft. AC: Formal analysis, Project administration, Supervision, Visualization, Writing – review & editing. EK: Formal analysis, Supervision, Writing – review & editing. MP: Data curation, Formal analysis, Writing – review & editing. SG: Formal analysis, Supervision, Writing – review & editing. IW: Formal analysis, Supervision, Writing – review & editing. EG: Formal analysis, Methodology, Project administration, Supervision, Writing – review & editing.
